# Cytomegalovirus retinitis in HIV/AIDS patients

**Published:** 2014-06-25

**Authors:** C Chiotan, L Radu, R Serban, C Cornăcel, M Cioboata, A Anghel

**Affiliations:** * Prof. Dr. Matei Bals" National Institute of Infectious Diseases Bucharest, Department of HIV / AIDS, Office of Ophthalmology; ** SCUOB; *** Ophthalmology Emergency Hospital, Bucharest

**Keywords:** CMV, cytomegalovirus retinitis, HIV/AIDS

## Abstract

Abstract

Human immunodeficiency virus (HIV) has the ability to affect any organ in the body. In 70% of HIV-infected patients ocular manifestations were observed, which in the vast majority reflect the systemic disease and may be the first sign of a disseminated infection.

Aim: The purpose of this paper is to determine the prevalence and the clinical aspects of cytomegalovirus retinitis in HIV/AIDS (Acquired Immunodeficiency Syndrome) patients.

Method: The study is retrospective, conducted in the Ophthalmology Office of "Matei Bals" Infectious Diseases Hospital in Bucharest during the period August 1, 2007 - August 1, 2013. Each patient was examined thoroughly at the slit lamp biomicroscope by using a lens of 90D and a 20D lens using the indirect microscope after administration of topical mydriatics.

Results: 131 patients were followed for HIV / AIDS with posterior segment ocular involvement. 36.64% of the 131 patients having affected the posterior segment have been diagnosed with CMV retinitis.

Conclusions: Doctors should be aware of the existence of ocular damage in HIV/AIDS and to emphasize the importance of regular ophthalmologic examination of patients with HIV/AIDS.

Abbreviations: HIV- human immunodeficiency virus, AIDS- acquired immunodeficiency syndrome, CMV retinitis- cytomegalovirus retinitis

## Introduction

HIV / AIDS is undoubtedly a multisystemic disease, but eye diseases occur in up to 70% of the cases during the natural history of infection. The spectrum of HIV-associated ophthalmic manifestations is very broad and extends from a simple blepharitis to blindness induced CMV retinitis (cytomegalovirus retinitis) [**1,3**].

 On June 30, 2012 there were a number of 11 189 patients of HIV/AIDS alive in Romania. This means that approximately 7,800 patients may have had the ocular manifestations of the disease [**4,5**].

 Ophthalmic pathology in those infected with HIV is due to opportunistic infections, vascular anomalies, neoplasms, diseases induced by specific medication or neuroophtalmic damage. Among these, opportunistic infections are the main cause of morbidity and ocular disease, with the highest potential for destruction in patients with AIDS [**6**].

 Coinfection with CMV occurs in 75-85% of the patients with HIV infection, of whom, more than a half develop CMV retinitis. Despite the high incidence, there are difficulties regarding the therapeutic approach and the results can only be satisfactory even with HAART highly active treatment [**2,7**].

 The incidence of retinal microangiopathy in patients with advanced HIV disease is related to the severity of the immunodeficiency and is a bad prognostic sign [**8,9**].

 Cytomegalovirus retinitis is the most common ocular opportunistic infection, representing 90% of the infectious retinitis, 20-30% of the patients with AIDS develop CMV retinitis. It usually occurs in the late stages of the disease (about 18 months after the declaration of the clinical onset) in patients with a lower limit of CD4 levels of 50/mmc [**10**].

 The ophthalmic disease usually occurs in patients presenting a clinically apparent systemic infection. It is always bilateral.

 Clinical forms:

 - A Typical form, edematous

 - B Atypical form, indolent

 - C Perivascular form

 - D Optic neuropathy

 A. Edematous form

 Early lesions appear as white spots, often centered on retinal vessels. They are accompanied by many hemorrhages, which is typical.

 Retinal lesions cause atrophic areas, partially pigmented. They made the image "in concarda" - outside a crown micro-outbreak point to remember, a viral proliferation and the central area of the retina atrophy vessels that have the appearance of white cords. The pathognomonic appearance has the following characteristics: at the periphery small white lesions, a viral proliferation zone and the central zone with atrophic retina with vessels of white cords appearance.

 B. Indolent form

 It occurs in cases of relapses under the maintenance therapy – a zone of thinned retina with diffuse pigmentation through which the choroid can be seen is described. The edge of the lesion is granulomatous, with small dots infiltrates, without hemorrhages. It is difficult to diagnose because it may appear as a scar lesion, but continues to progress slowly.

 C. Perivascular form – "frosted branch angiitis"

 It associates edematous retinitis with hemorrhages and tends to appear perivascularly. Periphlebitis may occur as an expression of retinitis that extends along vessels (it is not an inflammation of the vessels).

 D. Optic neuropathy occurs in 4% of all cases of CMV retinitis. The prognosis is bad, with frequent loss of light perception despite conventional antiretroviral therapy.

 In more than half of the patients, CMV retinitis is asymptomatic (54%), being revealed by systematic screening of the ocular fundus [**9,11,12**.

 When present, symptoms include decreased visual acuity, floaters that translate an evolved retinitis or an early involvement of the macula or of the optic nerve.

 The natural evolution represents the damage of the retina in 2-3 months. The most common complications are macular damage (with marked decrease in visual acuity), optic neuropathy, optic nerve atrophy, retinal detachment [**11,12**].

## Methods

 There were 48 patients with HIV/AIDS included in the study, who presented with CMV retinitis at the Ophthalmology Office of "Matei Bals" National Institute of Infectious Diseases Bucharest, from August 1, 2007 to August 1, 2012. No case has been taken from other authors or other hospitals of the same profile.

 The study was conducted retrospectively, records of all patients presenting with HIV / AIDS who experienced posterior segment ocular disease were used for data analysis.

 The examination sheet of each patient registered the age, sex, number of CD4 copies/μL at presentation, adherence to the treatment. Detailed ocular examination displays visual acuity measurement, examination of the anterior segment and posterior segment of the eye. After sampling the biological probes (blood, conjunctival discharge on sterile environment), the etiologic agent of posterior segment ocular manifestations could be established.

 Visual acuity was recorded in Snellen eye chart through Snellen fractions. The examination of the ocular posterior segment included thee examination of the vitreous and retina after administration of mydriasis - tropicamide 1% by using the 90D lens for the slit lamp microscope and the 20D lens for the indirect microscope for better visualization of the retinal periphery. Documentation of relevant changes at the vitreous and retinal examination was performed by fundus photography and fundus appearance diagrams.

 These individual data were analyzed and results were reported in percentage or absolute numerical value.

## Results

 CMV retinitis that occurred in 47 patients in the group, who had CD4 count <50/mL, maintained CMV as an opportunistic agent of the immunocompromised ones. One patient had a CD4 count of 198 copies / mL, indicating more likely that the CMV infection preceded the CD4 cell recovery.

 All the patients were submitted to an examination of the anterior pole without signs of inflammation, most presented with floaters, with decreased visual acuity gradually installed.

**Table 1** shows a preponderance of cases of CMV retinitis clinically manifested as edematous (60.41%), its pathognomonic appearance being an aid in the early detection of CMV retinal damage.

**Table 1:** Cases of CMV retinitis

Clinical form	Number of eyes
Edematous form	29
Indolent form	5
Perivascular form	10
Optic neuropathy	4

There were 2 cases of indolent forms which associated in the contralateral eye optic neuropathy, both of them with poor prognosis, to a total decrease of light perception despite the induction treatment that was started immediately.

 20.83% of the patients had clinical signs of the perivascular form; two of these patients had bilateral involvement, but were responsive to treatment.

 Prognosis proved poor for the two patients who had optic neuropathy in both eyes; unfortunately, both of them remained with low light perception, a vague light perception in one eye.

 Sustained specific anti CMV treatment (**Table 2**) stopped the progression of the retinitis in 38 patients. 

**Table 2:** Sustained specific anti CMV treatment

Response to treatment	Number of patients
Responsive patients	38
Nonresponsive patients	10

 Intravenous Ganciclovir or Foscarnet was chosen for systemic treatment at the first stage of induction, followed by the maintenance therapy with oral administration of Ganciclovir. Ganciclovir p.o. was administered until CD4 increased by 50 copies/μL above the level at which the presence of CMV retinitis was detected in asymptomatic patients and by 70 copies/μL in symptomatic patients. The treatment with oral ganciclovir was resumed only when the levels of CD4 decreased below 100 copies/μL or when the progression of retinal lesions was noted.

 The 38 patients who relapsed after the cessation of maintenance therapy can be called relatively stationary, but after another intravenous induction and maintenance therapy, a satisfactory visual acuity is kept.

 The induction therapy with oral ganciclovir was chosen for CMV retinitis with parafoveal involvement, nearest to the optic nerve or in the involvement of both eyes by CMV retinitis (peripheral lesions frequently).

 The treatment was maintained for a long time in order for the other eye or the viscera not to be involved.

 In 20.83% of the patients with CMV retinitis, the evolution could not be prevailed; they have developed relapses of retinal detachment after surgical treatment, optic neuropathy associated with CMV.

 4 of the 14 patients with binocular involvement presented with limited damaged retinal areas to the 8 patients who presented with total or partial retinal detachment, so at a drug exceeded stage of the disease with low or very low (light perception) visual acuity after vitreo-retinal surgery. Two other patients experienced inflammation impairment of the optic nerve, as shown in **Table 3**.

**Table 3:** Inflammation impairment of the optic nerve

Ocular involvement at presentation	Number of patients
Unilateral	25
Bilateral	14

## Conclusions

 The most common etiological agent for retinitis in HIV infected patients is cytomegalovirus (88.63% of retinitis in HIV/AIDS patients).

 Most patients had a CD4 lymphocyte count less than 50/μL, which can represent the susceptibility to this type of retinitis when lymphocyte count falls below this threshold.

 The edematous form was the most frequently encountered. The treatment consisted of systemic intravenous administration of Ganciclovir or Foscarnet at a first stage of induction, followed by the maintenance treatment with oral administration of Ganciclovir.

 The expected outcome in the management of CMV retinitis was to stop its progression, this being possible especially for the edematous form, the other types of CMV retinitis generally having a poor outcome, meaning that the visual function was severely impaired by various complications that might have arisen.
